# Canadian in-hospital mortality for patients with emergency-sensitive conditions: a retrospective cohort study

**DOI:** 10.1186/s12873-019-0270-1

**Published:** 2019-10-22

**Authors:** Simon Berthelot, Eddy S. Lang, Hude Quan, Henry T. Stelfox

**Affiliations:** 1 Axe Santé des populations et pratiques optimales en santé, Centre de recherche du CHU de Québec-Université Laval, 2705 Boul. Laurier, Québec, G1V 4G2 Canada; 2 Département de médecine familiale et de médecine d’urgence, Université Laval, 1050 avenue de la Médecine, Québec, Québec G1V 0A6 Canada; 3 Department of Emergency Medicine, Foothills Medical Centre, University of Calgary, 1403 29 Street NW, Calgary, Alberta T2N 2T9 Canada; 4 Department of Community Health Sciences, University of Calgary, TRW Building, 3280 Hospital Drive, Calgary, Alberta T2N 4Z6 Canada; 5 Department of Critical Care, University of Calgary and Alberta Health Services, McCaig Tower, 1403 29 Street NW, Calgary, Alberta T2N 2T9 Canada

**Keywords:** Emergency department, Mortality, Standardized mortality ratio, Emergency sensitive conditions

## Abstract

**Background:**

The emergency department (ED) sensitive hospital standardized mortality ratio (ED-HSMR) measures risk-adjusted mortality for patients admitted to hospital with conditions for which ED care may improve health outcomes. This study aimed to describe in-hospital mortality across Canadian provinces using the ED-HSMR.

**Methods:**

Hospital discharge data were analyzed from April 2009 to March 2012. The ED-HSMR was calculated as the ratio of observed deaths among patients with emergency-sensitive conditions in a hospital during a year (2010–11 or 2011–12) to the expected deaths for the same patients during the reference year (2009–10), multiplied by 100. The expected deaths were estimated using predictive models fitted from the reference year. Aggregated provincial ED-HSMR values were calculated. A HSMR value above or below 100 respectively means that more or fewer deaths than expected occurred within a province.

**Results:**

During the study period, 1,335,379 patients were admitted to hospital in Canada with an emergency-sensitive condition as the most responsible diagnosis. More in-hospital deaths (95% confidence interval) than expected were respectively observed for the years 2010–11 and 2011–12 in Newfoundland [124.3 (116.3–132.6); & 117.6 (110.1–125.5)] and Nova Scotia [116.4 (110.7–122.5) & 108.7 (103.0–114.5)], while mortality was as expected in Prince Edward Island [99.9 (86.5–114.8) & 100.7 (87.5–115.3)] and Manitoba [99.2 (94.5–104.1) & 98.3 (93.5–103.3)], and less than expected in all other provinces and territories.

**Conclusions:**

Our study revealed important variation in risk-adjusted mortality for patients admitted to hospital with emergency-sensitive conditions among Canadian provinces. The ED-HSMR may be a useful outcome indicator to complement existing process indicators in measuring ED performance.

**Trial registration:**

N/A – Retrospective cohort study.

## Background

On June 2016, Québec’s health and welfare commissioner published a report stating that the province had experienced years of the worst emergency department (ED) wait times in Canada and among high-income countries [1]. Two years later, Québec data on ED length of stay (LOS) are still concerning and in fact all Canadian provinces lag behind many other jurisdictions in their attempts to grapple with this important public health issue [1, 2]. A compelling body of evidence associates overcrowding and prolonged ED LOS with adverse effects such as increased time to thrombolysis [3]; delays in antibiotics administration [3–5] and pain management [3, 6, 7]; patient dissatisfaction [3]; and an increased in-hospital and out of hospital mortality [3, 8–10]. However, metrics of ED operations only capture part of the quality picture as they focus only on time-based quality measures and do not include patient outcomes that remain the foremost validation of the performance of health systems and the ultimate targets for quality improvement initiatives [11].

Assessing health outcomes in the ED setting represent a significant challenge as most ED-relevant outcomes are usually observed in other care settings (e.g. ICU, hospital ward or home care) where patients have been transferred after initial ED management. Notwithstanding the complexity of linking outcomes to ED care, most expert guidelines still recommend the inclusion of outcome measures, such as mortality, to a comprehensive ED performance assessment framework [12–14].

The hospital standardized mortality ratio for emergency department sensitive conditions (ED-HSMR) represents such a metric [15–17]. Adapted from a methodology used by the Canadian Institute for Health Information (CIHI) for tracking overall in-hospital mortality [18], the ED-HSMR specifically measures risk-adjusted mortality for patients admitted to hospital with emergency sensitive conditions, which are conditions where ED management may potentially improve outcomes. Using a consensus methodology [17] and a national survey of ED care providers [15], 92 potential emergency-sensitive conditions (e.g. ectopic pregnancy) were identified and evaluated. Among these conditions, 37 mortality-related emergency sensitive diagnosis groups (e.g., A41 sepsis) from the 10th Canadian version of the International Classification of Disease (ICD-10-CA) were used to develop the emergency care sensitive HSMR variant. Easily retrieved from administrative databases, the ED-HSMR has been shown to be valid and reliable [16]. It allows institutions or jurisdictions to follow their ED patient mortality over time and trigger internal performance reviews if trends are worrisome. It also provides opportunities for interprovincial comparisons of health outcomes observed among patients with emergency-sensitive conditions.

Consequently, to gain a broader understanding of ED performance and of the quality of care provided to emergency-sensitive conditions in Canada, this study aimed to describe in-hospital mortality across provinces using the ED-HSMR.

## Methods

### Study design and setting

We conducted a retrospective cohort study on national hospital discharge databases. We reviewed discharges from acute inpatient care institutions between April 1st 2009 and March 31st 2012 for nine provinces and three territories, and between April 1st 2009 and March 31st 2011 for the province of Québec. CIHI provided all administrative and patient-level data. Under the Canada Health Act, provinces and territories must provide universal health coverage to their citizens. All Canadian acute care facilities are publicly funded and owned.

### Hospital selection

We included all Canadian acute care facilities with an emergency department and hospital-based acute inpatient care. We excluded cancer centres, children’s hospitals and heart institutes because they treat specific populations with very different case-mixes. For risk-adjustment purpose, hospitals were classified into one of four peer-groups (teaching, large community, medium community and small community) based on academic designation, patient complexity and volume (see Additional file 1). This classification was adopted from the validated methodology used by the Canadian Institute for Health Information to calculate an all-cases HSMR [18].

### Case selection

We included all patients discharged dead or alive from hospital during the study period if they met the following criteria: 1) Admission to hospital through the ED; 2) Discharge from hospital with one of the 37 mortality-related emergency-sensitive diagnosis groups captured in the ED-HSMR (see Additional file 2); 3) Age between 29 days and 120 years at hospital admission; 4) Hospital length of stay equal to or less than 365 days; 5) Canadian resident. Patients were excluded if: 1) they were deceased at ED arrival; and 2) they were discharged against medical advice. Inclusion and exclusion criteria were directly derived from the methodology employed by CIHI to calculate the overall Canadian HSMR [18].

### Statistical analysis

#### Characteristics of the institutions and patients

Where it appropriately applied, medians and interquartile ranges, means or proportions with 95% confidence intervals were used to describe the characteristics of the institutions and of the cases included. Unadjusted mortality rates for each one of the 37 emergency-sensitive diagnosis groups included in the ED-HSMR were calculated per hospital and per year of the study period.

#### ED-HSMR calculation

ED-HSMRs were calculated for the fiscal years (April to March) 2010–2011 and 2011–2012 with the following equation:

$$ \frac{\mathrm{Actual}\ \mathrm{number}\ \mathrm{of}\ \mathrm{deaths}\ \mathrm{among}\ \mathrm{patients}\ \mathrm{with}\ \mathrm{emergency}\hbox{-} \mathrm{sensitive}\ \mathrm{diagnosis}\ \mathrm{groups}\ \mathrm{in}\ 2010\hbox{-} 11\ \mathrm{or}\ 2011\hbox{-} 12}{\mathrm{Expected}\ \mathrm{number}\ \mathrm{of}\ \mathrm{deaths}\ \mathrm{among}\ \mathrm{same}\ \mathrm{patients}\ \mathrm{based}\ \mathrm{on}\ \mathrm{mortality}\ \mathrm{probabilities}\ \mathrm{in}\ \mathrm{the}\ \mathrm{reference}\ \mathrm{year}\left(2009\hbox{-} 10\right)}\times 100 $$

A 95% confidence interval was calculated for each HSMR value using the Byar’s approximation [18]. A HSMR value above 100 means that more deaths than expected occurred in an acute care facility. Conversely, a HSMR value below 100 means fewer deaths than expected occurred in an acute care facility. In other words, an ED-HSMR of 114 would indicate that each admission to a specific hospital for an emergency-sensitive condition has a probability of death 14% higher than what is expected from the Canadian average.

#### Expected deaths

We estimated the expected number of deaths in 2010–11 or 2011–12 using fixed effects logistic regression models derived from the reference year (2009–10) for each hospital-peer group. After modelling mortality with different independent variables, the following covariates were retained in the final ED-HSMR predictive models: diagnosis groups, age (continuous), gender (dichotomous), in-hospital length of stay (6 groups: 1, 2, 3–9, 10–15, 16–21 and 22–365 days) and comorbidities (3 groups based on Charlson index score: Group 0 = score 0 (outside Québec) or scores 0 and 1 (Québec); Group 1 = scores 1 and 2 (outside Québec) or scores 2, 3 and 4 (Québec); Group 2 = scores 3 and more (outside Québec) or scores 5 and more (Québec); see Additional file 3). For managing missing data, we used a single imputation method, assigning most frequent values for categorical variables and medians, for continuous variables.

Probability of death at patient-level was calculated using the appropriate hospital-peer group specific model (teaching, large community, medium community and small community) from the reference year. After conversion from the log odds of death (p_death_ = e^log odds of death^ / [1 + (e^log odds of death^)]), all individual patient probabilities were summed to get the expected number of deaths in a specific hospital, in 2010–11 or 2011–12. An ED-HSMR for a specific acute care facility was only calculated if more than 20 deaths were expected within the study year at the institution, as fewer deaths yield unreliable and volatile HSMR measures [19].

The discriminatory power and calibration of the risk-adjustment models used to estimate the expected number of deaths for each hospital were reported in a previous publication [16]. Areas under receiver operating characteristic curves of the predictive models used in the ED-HSMR were 0.80, 0.80, 0.80 and 0.81 for the teaching, large-community, medium-community and small-community peer-group hospitals, respectively.

#### Hospital-level and aggregated provincial ED-HSMRs

We report hospital-level ED-HSMRs through tables and caterpillar plots. Stratifications by peer-groups and provinces are graphically represented. Aggregated provincial ED-HSMR values were calculated by dividing the sum all observed (O) deaths with the sum of all expected (E) number of deaths of all institutions of a province or a territory (O/E × 100). All patients were included in aggregated measures, even those from hospitals with less than 20 expected deaths where no site-specific HSMR could be estimated. Analyses were performed using Stata version MP 11.2 (StataCorp, TX, USA).

## Results

### Characteristics of cases and hospitals

During the 3-year study period, 1,335,379 patients were admitted to 629 hospitals across 11 provinces and territories from the ED with one of the 37 mortality-related emergency sensitive diagnosis groups captured in the ED-HSMR as the most responsible diagnosis. Table 1 describes their characteristics. Half of the cohort was composed of females older than 73 years and hospitalized for 5 days or less. Our study population presented a low comorbidity burden as more than 80% of patients had a Charlson index score of 0 or 1. Small hospitals represented 58% of all hospitals included in our study, but treated only 10.8% of all patients admitted. Hospital distribution was markedly different in the province of Québec where 42.5% of all hospitals were either teaching or large-community institutions. Provinces and Territories in the rest of Canada had a higher proportion of small-community hospitals (Table 2).

**Table 1:** Characteristics of patients* (*n* = 1,335,379)^a^

Median Age (IQR)	73 (59–83)
Male	673,102 (50.4)
Charlson score	
0	852,794 (63.9)
1	226,960 (17.0)
2	139,624 (10.5)
3	59,522 (4.5)
4	17,885 (1.3)
≥ 5	38,594 (2.9)
Median In-hospital length of stay [days (IQR)]	5 (3–11)
Transfer from another acute care facility to ED	35,290 (2.6)
In-hospital deaths	118,649 (8.9)
Hospital peer-groups	
Teaching	326,585 (24.5)
Community - Large	583,776 (43.7)
Community - Medium	280,549 (21.0)
Community - Small	144,469 (10.8)
Province^b^	
Ontario	542,335 (40.6)
Québec	210,914 (15.8)
British Columbia	195,956 (14.7)
Alberta	140,809 (10.5)
Saskatchewan	59,019 (4.4)
Manitoba	55,921 (4.2)
New Brunswick	44,236 (3.3)
Nova Scotia	44,001 (3.3)
Newfoundland and Labrador	30,277 (2.7)
Prince Edward Island	7661 (0.6)
Territories	4250 (0.3)

^*^All data are presented as number and percentage [n (%)] unless otherwise indicated

^a^Cohort composed of patients admitted with one of 37 emergency-sensitive Diagnosis Groups for whom ED care may reduce in-hospital mortality, as identified with a multidisciplinary panel (Berthelot et al. 2014)

^b^Data from fiscal years 2009–10, 2010–11 and 2011–12, except Québec (2009–10 and 2010–11)

**Table 2:** Number of hospitals (*N* = 629) per peer-group and province*

Province	Teaching	Community-Large	Community-Medium	Community-Small	*Total*
Newfoundland and Labrador	1 (3.5)	0 (0)	5 (17.2)	23 (79.3)	29
Prince Edward Island	0 (0)	1 (20.0)	1 (20.0)	3 (60.0)	5
Nova Scotia	1 (3.1)	1 (3.1)	8 (25.0)	22 (68.8)	32
New Brunswick	1 (5.0)	4 (20.0)	5 (25.0)	10 (50.0)	20
Québec	16 (17.0)	24 (25.5)	28 (29.8)	26 (27.7)	94
Ontario	13 (8.1)	35 (21.9)	38 (23.8)	74 (46.3)	160
Manitoba	2 (3.4)	5 (8.5)	7 (11.9)	45 (76.3)	59
Saskatchewan	5 (8.3)	0 (0)	6 (10.0)	49 (81.7)	60
Alberta	3 (3.2)	8 (8.6)	6 (6.5)	76 (81.7)	93
British Columbia	2 (2.8)	15 (21.1)	20 (28.2)	34 (47.9)	71
Territories	0 (0)	0 (0)	2 (33.3)	4 (66.7)	6
*Total*	44	93	126	366	629

^*^All data are presented as number and percentage of provincial hospital coverage [n (%)]

### Overall and diagnosis group specific mortality rates

For the 3 years of the study period (including the reference year), overall mortality in our cohort was 8.9%, with chronic obstructive pulmonary disease, pneumonia, heart failure, acute myocardial infarction, stroke/cerebral infarction, and sepsis accounting for 55.5% of all deaths (Table 3). Diagnosis groups with the highest mortality rates were cardiac arrest (65.1%) and shock not elsewhere classified (50.0%).

**Table 3:** Study population (*N* = 1,335,379) distribution and mortality rates by Diagnosis Group

Diagnosis Groups included in the ED-HSMR		No of Patients	% of Patients	Number of deaths	Mortality rate (%)	Mortality rate 95% CI	
J44	Other chronic obstructive pulmonary disease	172,451	12.9	11,828	6.9	6.7	7.0
J18	Pneumonia	137,704	10.3	11,674	8.5	8.3	8.6
I50	Heart failure	130,597	9.8	13,393	10.3	10.1	10.4
I21	Acute Myocardial Infarction (AMI)	121,463	9.1	8983	7.4	7.2	7.5
S72	Fracture of femur	81,591	6.1	4325	5.3	5.1	5.5
K56	Paralytic ileus and intestinal obstruction without hernia	69,397	5.2	2558	3.7	3.5	3.8
I63	Cerebral infarction	50,987	3.8	6472	12.7	12.4	13.0
K85	Acute pancreatitis	46,149	3.5	714	1.5	1.4	1.7
E11	Diabetes Mellitus type 2	43,269	3.2	1672	3.9	3.7	4.0
L03	Cellulitis	41,177	3.1	645	1.6	1.4	1.7
A41	Sepsis	40,769	3.1	10,198	25.0	24.6	25.4
K57	Diverticular disease of intestine	38,133	2.9	633	1.7	1.5	1.8
K92	Other diseases of digestive system	34,946	2.6	1639	4.7	4.5	4.9
N17	Acute renal failure	31,631	2.4	3612	11.4	11.1	11.8
S06	Intracranial injury	29,756	2.2	3294	11.1	10.7	11.4
E87	Other disorders of fluid, electrolyte and acid-base balance	25,760	1.9	690	2.7	2.5	2.9
S32	Fracture of lumbar spine and pelvis	24,063	1.8	681	2.8	2.6	3.0
I64	Stroke, not specified as haemorrhage or infarction	23,687	1.8	3328	14.0	13.6	14.5
I26	Pulmonary embolism	23,237	1.7	1335	5.7	5.4	6.0
J69	Pneumonitis due to solids and liquids	20,131	1.5	5747	28.5	27.9	29.2
F05	Delirium, not induced by alcohol and other psychoactive substances	20,038	1.5	1215	6.1	5.7	6.4
I24	Other acute ischemic heart disease	17,864	1.3	606	3.4	3.1	3.7
E86	Volume depletion	13,475	1.0	634	4.7	4.3	5.1
J96	Respiratory failure, not elsewhere classified	12,351	0.9	4365	35.3	34.5	36.2
K55	Vascular disorders of intestine	11,554	0.9	1791	15.5	14.8	16.2
K26	Duodenal ulcer	10,964	0.8	565	5.2	4.7	5.6
I61	Intracerebral haemorrhage	10,783	0.8	3440	31.9	31.0	32.8
T82	Complications of cardiac and vascular prosthetic devices, implants and grafts	9182	0.7	362	3.9	3.5	4.3
K72	Hepatic failure	7040	0.5	1231	17.5	16.6	18.4
I62	Other non traumatic intracranial haemorrhage	5467	0.4	1141	20.9	19.8	21.9
K65	Peritonitis	5433	0.4	450	8.3	7.5	9.0
R57	Shock, not elsewhere classified	5130	0.4	2566	50.0	48.7	51.4
I71	Aortic aneurism and dissection	5033	0.4	1277	25.4	24.2	26.6
I60	Subarachnoid haemorrhage	4797	0.4	1043	21.7	20.6	22.9
G93	Other disorders of brain	4179	0.3	1648	39.4	38.0	40.9
I46	Cardiac arrest	3582	0.3	2331	65.1	63.5	66.6
J80	Adult respiratory distress syndrome	1609	0.1	563	35.0	32.7	37.3
	All conditions	1,335,379	100.0	118,469	8.9	8.8	8.9

### Hospital-level ED-HSMRs

The ED-HSM was estimated for 46.7 and 33.9% (Québec data not available) of all Canadian hospitals in 2010 and 2011, respectively (see Additional files 4 and 5). Among hospitals with sufficient number of expected deaths to calculate the ED-HSMR, 22.8% in 2010 and 31.5% in 2011 had a HSMR 95% confidence interval falling below the 100 threshold (fewer deaths than expected), and 10.5% in 2010 and 7.0% in 2011 had a HSMR 95% confidence interval falling above (more deaths than expected). Results are stratified by province and hospital-peer group in Table 4.

**Table 4:** Number of Hospitals with an ED-HSMR 95% confidence interval falling under or over 100 by peer-group and province in 2010 (*N* = 294) and 2011 (*N* = 213)*

	2010			2011		
	*< 100*	*> 100*	*Total*	*< 100*	*> 100*	*Total*
Peer-Groups						
*Teaching*	14 (33.3)	6 (14.3)	42	11 (44.0)	1 (4.0)	25
*Large Community*	31 (33.3)	8 (8.6)	93	35 (50.7)	2 (2.9)	69
*Medium Community*	22 (18.0)	10 (8.2)	122	18 (18.9)	9 (9.5)	95
*Small Community*	0 (0)	7 (18.9)	37	3 (12.5)	3 (12.5)	24
Provinces						
*Newfoundland/Labrador*	0 (0)	5 (71.4)	7	0 (0)	4 (50.0)	8
*Prince Edward Island*	0 (0)	0 (0)	2	0 (0)	0 (0)	2
*Nova Scotia*	0	5 (71.4)	7	0(0)	2 (20.0)	10
*New Brunswick*	3 (27.3)	0 (0)	11	4 (36.4)	0 (0)	11
*Québec*^*a*^	19 (25.3)	9 (12.0)	75	N/A	N/A	N/A
*Ontario*	24 (22.3)	9 (8.3)	105	38 (39.6)	6 (6.3)	96
*Manitoba*	0 (0)	0 (0)	12	0 (0)	1 (8.3)	12
*Saskatchewan*	3 (25.0)	1 (8.3)	12	4 (33.3)	1 (8.3)	12
*Alberta*	7 (38.9)	2 (11.1)	18	6 (30.0)	1 (5.0)	20
*British Columbia*	11 (28.2)	0 (0)	39	15 (36.6)	0 (0)	41
*Territories*^*b*^	0 (0)	0 (0)	0	0 (0)	0 (0)	1

*All data are presented as number and percentage [n (%)]

^a^Québec data not available in 2011

^b^No hospital in the Territories in 2010 met the criteria of at least 20 expected deaths for the calculation of the ED-HSMR

### Provincial aggregated ED-HSMRs

**Fig. 1:**
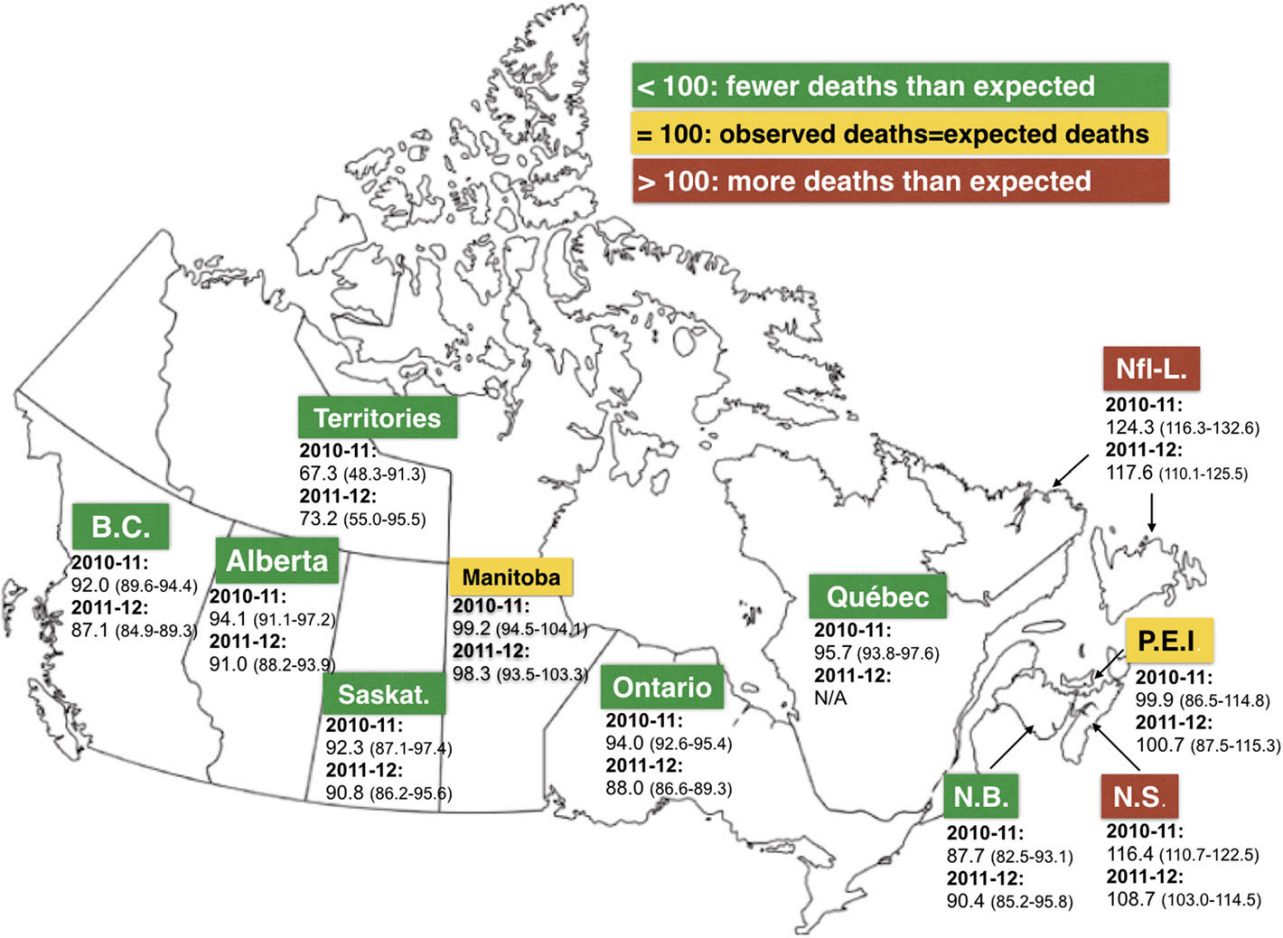
ED-HSMR (95% confidence interval) by province and territories for years 2010–11 and 2011–12. Green: < 100 = Fewer deaths than expected. Yellow: =100 = Observed deaths are equal to expected deaths. Red: > 100 = More deaths than expected

Figure 1 illustrates the aggregated ED-HSMR estimates by province for 2010–11 and 2011–12. In both years, Nova Scotia and Newfoundland-Labrador had provincial ED-HSMRs higher than 100, while the 95% confidence intervals of the aggregated measures for Prince Edward Island and Manitoba crossed the 100 threshold. All other provinces and territories experienced fewer deaths than what was expected from mortality trends recorded in Canada during the reference year (2009–10).

## Discussion

We analyzed adjusted mortality rates in Canada using the ED-HSMR. Our results show that there is significant interhospital and interprovincial variation in mortality trends for patients admitted with emergency-sensitive conditions and that this variation appears to be consistent over the 2 years studied. The ED-HSMR could be used to measure the performance of Canadian hospital systems in emergency care and guide quality improvement initiatives.

To the best of our knowledge, we are the first to report on variations in mortality across Canadian provinces for a comprehensive list of emergency sensitive conditions. Two recent studies published in 2017 have reported mortality trends in Canada for two domains overlapping with emergency medicine: trauma and acute myocardial infarction (AMI) care. Both studies pointed out interprovincial disparities in the management of these acutely ill populations. Tran et al. examined Canadian and provincial 30-day in-hospital mortality rates after percutaneous coronary interventions (PCI) and coronary artery bypass grafting (CABG) between 2004 and 2013. Ten-year adjusted odds (2004–2013) of mortality were higher after PCI in Saskatchewan and after CABG in Newfoundland and Labrador [20]. Similarly, Moore et al. reviewed data on 78,707 trauma patients in 7 provinces from 2006 to 2012. Authors reported that Nova Scotia, Newfoundland and Labrador, and Manitoba were the provinces with the highest risk-adjusted mortality rates in 2011 and 2012 [21]..

Our analyses yielded similar results with Newfoundland/Labrador, Nova Scotia, Manitoba and Prince Edward Island having the highest ED-HSMR estimates. Although our composite mortality indicator captures some trauma-related diagnoses (e.g. intracranial injury) and AMI, it is meant to employ a broad approach for identifying mortality trends involving 37 emergency sensitive conditions (e.g. sepsis). As a result, trauma and AMI care may explain a part of, but not all observed disparities reported in our own study. Moreover, mortality for emergency sensitive conditions appears to be higher than expected in provinces where the proportion of the population living in rural communities is highest [22]. This finding echoes previous reports that highlighted significant disparities in access to high-quality emergency care in Canadian rural communities [23, 24]. In the same way it was done for trauma care across the country, Canadian decision- and policy-makers could use the ED-HSMR to trigger in-depth performance assessment to help identify opportunities for improving emergency care structures and processes to improve patient outcomes.

Since 2005, CIHI calculates annually for all Canadian hospitals a HSMR based on 72 diagnosis groups (e.g. Alzheimer’s disease) explaining 80% of all Canadian in-hospital deaths. Although using the same methodology, our HSMR variant differs significantly from CIHI’s by capturing only those conditions for which ED care may potentially influence mortality (e.g. pulmonary embolism). Many reports have advocated caution in using HSMRs or any other mortality indicators [19, 25]. Incomplete risk adjustment, low rate of preventable deaths and inconstant concordance between mortality and other quality indicators are some of the limitations impeding inference we could draw from mortality metrics to potential quality of care breaches. Moreover, in-hospital mortality is usually remote from the ED and occurs on hospital wards, making it virtually impossible to completely isolate ED influence on patient outcomes. However, as imperfect as it may be, other reports have shown that tracking mortality rates can drive system-level changes for improving patient care and healthcare organizations [26–30]..

Similar problems arise with ED wait time metrics. Like mortality rates, they are reflections of a care system rather than processes of care specific to the ED. Blocked access to in-hospital beds, limited primary care resources for management of patients with multimorbidities and a lack of patient education on appropriate use of EDs are some of the factors that can impact ED flow, but are not caused per se by poor ED care. As for ED wait time metrics, the ED-HSMR could serve as a strong incentive for hospital departments and community health care resources to move from siloed approached to quality improvement and work together to improve outcomes of patients with emergency sensitive conditions.

The Québec’s health and welfare commissioner report published in 2016 raises important questions. As worrisome as they are, wait times henceforth do not appear to be the only important risk factor impacting outcomes of ED patients with emergency sensitive conditions. Indeed, Québec ED-HSMR in 2010–11 reveals fewer deaths than expected. This complementary information highlights the importance of synergistically using structure, process and outcome metrics when evaluating health care quality. We believe that all jurisdictions should adopt a comprehensive quality framework that would include among other metrics the ED-HSMR.

## Limitations

This study has inherent limitations of all studies using administrative database sources. Previous reports have shown that coding of administrative data is usually accurate and inaccuracies had modest effect on an HSMR [31–33]. The ED-HSMR does not adjust for all potential confounders, such as severity of disease, smoking habits or socio-economic status. Although severity of disease and smoking habits are not easily extractable from existing hospital databases, socio-economic status can be assessed using deprivation index derived from patient postal codes. Future iteration of the ED-HSMR could test socio-economic status as a potential explanatory variable. Furthermore the ED-HSMR is not a performance measure suitable for small hospitals because of the low numbers of observed and expected in-hospital deaths. Small hospitals represent 58% of all Canadian hospitals. Further research is needed to identify outcome measures that could be used to assess the quality of care provided in low-volume institutions. Finally, we acknowledge that our data are several years old. However, we believe that our results demonstrate the feasibility of using an ED sensitive condition hospital standardized mortality ratio to measure ED performance as a way to supplement existing wait times and access-to-care indicators.

## Conclusion

In conclusion, we analyzed Canadian in-hospital mortality rates using the ED-HSMR. Our study revealed important variation in risk-adjusted mortality for patients admitted to hospital with emergency-sensitive conditions among Canadian provinces. These results warrant in-depth evaluations to understand the root causes of the observed regional variation. The ED-HSMR may be a useful outcome indicator to complement existing process indicators in measuring ED performance.

## Supplementary information

**Additional file 1.** Hospital peer-groups definition

**Additional file 2. **List of the Diagnosis Groups (*n* = 37) of the International Classification of Diseases (10th version) included in the ED-HSMR

**Additional file 3.** Charlson index score groups

**Additional file 4. **Distribution of ED-HSMRs of all eligible institutions (*n* = 294) in 2010–11

**Additional file 5.** Caterpillar plots of ED-HSMRs by peer-group

## Data Availability

The datasets generated and analysed during the current study are not publicly available. Data were provided by the Canadian Institute for Health Information with an agreement to destroy in whole all datasets after study completion.

## Abbreviations

CIHI: Canadian Institute for Health Information
ED: Emergency department
ED-HSMR: Hospital standardized mortality ratio for emergency department sensitive conditions
ICU: Intensive care unit
LOS: Length of stay
