# Supplementary material for: Canadian in-hospital mortality for patients with emergency-sensitive conditions: a retrospective cohort study
Source: BMC Emerg Med. 2019 Oct 22;19:57. doi: 10.1186/s12873-019-0270-1 (PMC6805639; doi:10.1186/s12873-019-0270-1)
Supplement: Supplementary file 1 — Additional file 1. Hospital peer-groups definition [file 12873_2019_270_MOESM1_ESM.docx]

**Additional file 1. Hospital peer-groups definition**

| **Teaching** | Hospitals with full membership to the Association of Canadian Academic Healthcare Organizations (ACAHO) or teaching hospitals according to the Association québécoise des établissements en santé et services sociaux (AQESS) |
| --- | --- |
| **Community - Large** | 2 of the following 3 criteria:   - ≥ 8000 inpatient cases - ≥ 10 000 weighted cases - ≥ 50 000 inpatient days |
| **Community - Medium** | Do not meet large-community group criteria  ≥ 2000 weighted cases (approximately ≥ 50 beds) |
| **Community - Small** | Do not meet large-community group criteria  < 2000 weighted cases (approximately < 50 beds) |

Reproduced from : CIHI. Hospital Standardized Mortality Ratio (HSMR): Technical Notes. Public Release. Ottawa, February 2012.
