# Supplementary material for: Canadian in-hospital mortality for patients with emergency-sensitive conditions: a retrospective cohort study
Source: BMC Emerg Med. 2019 Oct 22;19:57. doi: 10.1186/s12873-019-0270-1 (PMC6805639; doi:10.1186/s12873-019-0270-1)
Supplement: Supplementary file 2 — Additional file 2. List of the Diagnosis Groups (n = 37) of the International Classification of Diseases (10th version) included in the ED-HSMR [file 12873_2019_270_MOESM2_ESM.docx]

**Additional file 2. List of the Diagnosis Groups (n=37) of the International Classification of Diseases (10^th^ version) included in the ED-HSMR**

| **A41** | Sepsis |
| --- | --- |
| **E11** | Diabetes Mellitus type 2 |
| **E86** | Volume depletion |
| **E87** | Other disorders of fluid, electrolyte and acid-base balance |
| **F05** | Delirium, not induced by alcohol and other psychoactive substances |
| **G93** | Other disorders of brain |
| **I21** | Acute Myocardial Infarction (AMI) |
| **I24** | Other acute ischemic heart disease |
| **I26** | Pulmonary embolism |
| **I46** | Cardiac arrest |
| **I50** | Heart failure |
| **I60** | Subarachnoid haemorrhage |
| **I61** | Intracerebral haemorrhage |
| **I62** | Other non traumatic intracranial haemorrhage |
| **I63** | Cerebral infarction |
| **I64** | Stroke, not specified as haemorrhage or infarction |
| **I71** | Aortic aneurism and dissection |
| **J18** | Pneumonia |
| **J44** | Other chronic obstructive pulmonary disease |
| **J69** | Pneumonitis due to solids and liquids |
| **J80** | Adult respiratory distress syndrome |
| **J96** | Respiratory failure, not elsewhere classified |
| **K26** | Duodenal ulcer |
| **K55** | Vascular disorders of intestine |
| **K56** | Paralytic ileus and intestinal obstruction without hernia |
| **K57** | Diverticular disease of intestine |
| **K65** | Peritonitis |
| **K72** | Hepatic failure |
| **K85** | Acute pancreatitis |
| **K92** | Other diseases of digestive system |
| **L03** | Cellulitis |
| **N17** | Acute renal failure |
| **R57** | Shock, not elsewhere classified |
| **S06** | Intracranial injury |
| **S32** | Fracture of lumbar spine and pelvis |
| **S72** | Fracture of femur |
| **T82** | Complications of cardiac and vascular prosthetic devices, implants and grafts |
