# Supplementary material for: Canadian in-hospital mortality for patients with emergency-sensitive conditions: a retrospective cohort study
Source: BMC Emerg Med. 2019 Oct 22;19:57. doi: 10.1186/s12873-019-0270-1 (PMC6805639; doi:10.1186/s12873-019-0270-1)
Supplement: Supplementary file 3 — Additional file 3. Charlson index score groups [file 12873_2019_270_MOESM3_ESM.docx]

**Additional file 3. Charlson index score groups**

| **Charlson Group** | **Charlson score** | |
| --- | --- | --- |
|  | **Outside Québec** | **In Québec** |
| **0** | 0 | 0 and 1 |
| **1** | 1 and 2 | 2, 3 and 4 |
| **2** | ≥ 3 | ≥ 5 |

Reproduced from : CIHI. Hospital Standardized Mortality Ratio (HSMR): Technical Notes. Public Release. Ottawa, February 2012.
