# Supplementary material for: Canadian in-hospital mortality for patients with emergency-sensitive conditions: a retrospective cohort study
Source: BMC Emerg Med. 2019 Oct 22;19:57. doi: 10.1186/s12873-019-0270-1 (PMC6805639; doi:10.1186/s12873-019-0270-1)
Supplement: Supplementary file 5 — Additional file 5. Caterpillar plots of ED-HSMRs by peer-group [file 12873_2019_270_MOESM5_ESM.docx]

**Additional file 5. Caterpillar plots of ED-HSMRs by peer-group**

**Additional file 5. (Continued)**
